# Comparing behavioral risk assessment strategies for quantifying biosecurity compliance to mitigate animal disease spread

**DOI:** 10.3389/fvets.2022.962989

**Published:** 2022-10-03

**Authors:** Eric M. Clark, Scott C. Merrill, Luke Trinity, Tung-Lin Liu, Aislinn O'Keefe, Trisha Shrum, Gabriela Bucini, Nicholas Cheney, Ollin D. Langle-Chimal, Christopher Koliba, Asim Zia, Julia M. Smith

**Affiliations:** ^1^Social Ecological Gaming and Simulation Lab, University of Vermont, Burlington, VT, United States; ^2^Department of Plant and Soil Science, University of Vermont, Burlington, VT, United States; ^3^Gund Institute for Environment, University of Vermont, Burlington, VT, United States; ^4^Computational Biology Research and Analytics Lab, University of Victoria, Victoria, BC, Canada; ^5^Department of Community Development and Applied Economics, University of Vermont, Burlington, VT, United States; ^6^Department of Computer Science, University of Vermont, Burlington, VT, United States; ^7^Complex Systems Center, University of Vermont, Burlington, VT, United States; ^8^Department of Animal and Veterinary Sciences, University of Vermont, Burlington, VT, United States

**Keywords:** experimental games, livestock disease, decision making, computer science, experimental economics, data science, computational social science

## Abstract

Understanding the impact of human behavior on the spread of disease is critical in mitigating outbreak severity. We designed an experimental game that emulated worker decision-making in a swine facility during an outbreak. In order to combat contamination, the simulation features a line-of-separation biosecurity protocol. Participants are provided disease severity information and can choose whether or not to comply with a shower protocol. Each simulated decision carried the potential for either an economic cost or an opportunity cost, both of which affected their potential real-world earnings. Participants must weigh the risk infection vs. an opportunity cost associated with compliance. Participants then completed a multiple price list (MPL) risk assessment survey. The survey uses a context-free, paired-lottery approach in which one of two options may be selected, with varying probabilities of a high and low risk payouts. We compared game response data to MPL risk assessment. Game risk was calculated using the normalized frequency of biosecurity compliance. Three predominant strategies were identified: risk averse participants who had the highest rate of compliance; risk tolerant participants who had the lowest compliance rate; and opportunists who adapted their strategy depending on disease risk. These findings were compared to the proportion of risk averse choices observed within the MPL and were classified into 3 categories: risk averse, risk tolerant and neutral. We found weak positive correlation between risk measured in our experimental game compared to the MPL. However, risk averse classified participants in the MPL tended to comply with the biosecurity protocol more often than those classified as risk tolerant. We also found that the behavioral risk clusters and categorization *via* the MPL were significantly, yet weakly associated. Overall, behavioral distributions were skewed toward more risk averse choices in both the MPL and game. However, the MPL risk assessment wasn't a strong predictor for observed game behavior. This may indicate that MPL risk aversion metrics might not be sufficient to capture these simulated, situational risk aversion behaviors. Experimental games have a large potential for expanding upon traditional survey instruments by immersing participants in a complex decision mechanism, and capturing dynamic and evolving behavioral signals.

## 1. Introduction

Understanding how human behavior impacts the spread of disease is crucial in strengthening agricultural industries. Our research focuses on building interactive games and simulations to emulate complex decision-mechanisms that can impact the well-being of agricultural production networks. This can be especially useful for testing how people respond to risk communication strategies in preparation for infectious disease outbreaks. Here, we compare risk classifications using a traditional survey-based risk assessment to response data collected using an experimental game with the goal of helping to inform decision support during potential disease outbreak scenarios.

In order to combat disease spread, biosecurity protocols and the practices that they detail have become prominent tools, integrated into standard operating procedures among the agricultural industry (1, 2). These may include forms of decontamination like truck washes, line of separation protocols, and other disease preventative measures like vaccinations, and feed treatment. However, the use and compliance with biosecurity protocols implemented within farms can vary (3). Some of these procedures may be onerous or cumbersome and at times bypassed by workers (4, 5). Motivators of biosecurity decision making are unclear, but decision ramifications can be substantial.

Experimental gaming simulations can be used as digital tools for studying factors that influence behavior and decision-making, including in various sectors of agriculture (6–12). Since risk communication is an important tool for education and nudging behaviors for crisis mitigation (13), several of our experimental games were designed to test how various risk communication strategies impact decision-making. In another experimental game, we simulated biosecurity investment amidst several outbreak scenarios along pig production supply chains (6). This allowed us to identify prominent strategies and behaviors that were used to address conflicts associated with disease in agriculture (9, 10). In Clark et al. (9), clustering algorithms (14) were shown to be a useful tool for categorizing behavioral observations from the experimental game. We've also shown how non-monetary awards and incentives can affect conservation decisions in farms (11). Experimental gaming simulations can be applied to quantify situational risk aversion associated with agricultural decision-making. This may be especially useful for identifying and accounting for biosecurity non-compliance among worker populations within agricultural production networks.

There are many avenues used to assess behavior associated with confronting risk. One such tool ,MPL risk assessment (15, 16), has shown potential in quantifying economic risk preferences. We compare our simulation results with a well-tested MPL risk assessment strategy, Holt and Laury (16) in which 10 disjoint binary lotteries are presented, and participants choose between a safe or risky option. The payouts are structured such that the probability of success for choosing the more risky, higher paying option sequentially increases. Participants generally move from the less to more risky option as the probability spread between the two choices becomes more favorable. The proportion of safe choices, and the probability point at which participants switch from the safe to risky option can provide a metric for comparing risk aversion profiles. This general context-free approach has been correlated to real world behaviors in finance (17). Risk aversion *via* the MPL was found to be associated with less participation in negative health practices, including smoking, excessive alcohol consumption, and lack of seatbelt compliance (18). Since the MPL-style risk aversion metrics are context-free, it is unclear how context-related conflict will influence behavior, given the possibility that risk aversion may evolve throughout an individual's experience with a complex decision mechanism.

Our study uses an experimental game to emulate potential rule breaking behavior (i.e, non-compliance) with a line of separation shower protocol within a pig production facility. The aim of our simulation is to understand under what circumstances participants will engage in risky behaviors during an outbreak scenario. We then compared observed behavioral risk to a well-tested MPL risk assessment survey. We hypothesized that risk aversion observed within the experimental simulation would correspond to risk aversion measured in the MPL assessment.

## 2. Methods

The experimental game was originally designed and featured in Merrill et al. (7). An additional, larger sample of online participants was recruited for this follow-up study. The game emulates working in a pig production facility during an infectious disease outbreak. To begin the experiment, participants engaged with an introductory slide show to provide contextual background information and frame the simulated decision mechanism. Participants then completed a practice round, featuring an interactive tutorial, in order to acquaint them with the simulation mechanics. These practices were accepted by the University of Vermont Institutional Review Board concerning experiments using human participants (University of Vermont IRB # CHRBSS-16-232-IRB).

The experimental game was designed using Unity game development software (Unity Technologies, Version 5.6.3) and was built for online deployment using webGL (19). Data were compiled using a relational database. Participants were recruited using the Amazon Mechanical Turk (MTurk) survey marketplace (20–22). We motivated participants with performance based incentives, as a means to bolster immersion and increase effort throughout the experiment (23). Player earnings from the experimental simulation were converted at a rate of $350 simulation dollars to $1 USD. A demo of the experimental game is hosted here: https://segs.w3.uvm.edu/demos/compliance/.

**Figure 1:**
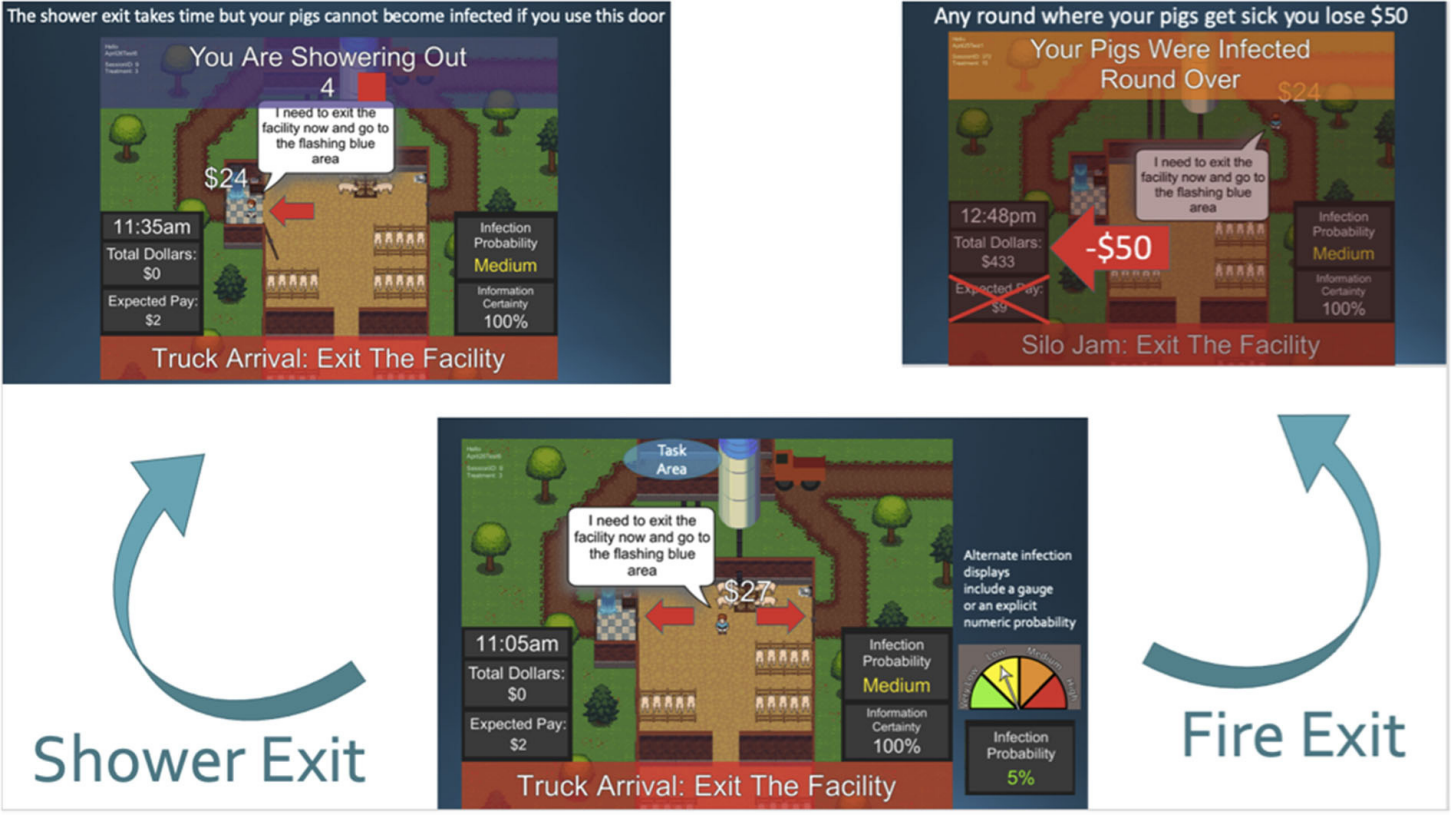
Screenshot of the compliance game decision mechanism. Participants can choose to comply with a biosecurity shower protocol carrying an associated time cost, or bypass *via* the emergency exit which can subject the facility to infection.

During the game, participants controlled a digital avatar throughout several simulated workdays. Participants were directed to collect coins, which symbolized indoor tasks, as well as completion of a single outdoor task per day. When the outdoor task was initiated, a score timer began ticking down (see Figure 1). The player then decided to either comply with the shower-in shower-out biosecurity practice or exit *via* the emergency exit. The emergency exit was faster (and thus more profitable), but risked the possibility that the animals in the facility could become infected with a disease. Choosing the shower-in, shower-out biosecurity practice exit effectively prevented animals from becoming sick. However, the shower-in, shower-out practice required that the participant wait 5 s before both exiting and returning to the facility. The time spent “showering” resulted in lost potential earnings both with the outdoor task losing value, and with not being able to collect coins (as inside tasks) upon re-entering the facility. Using observations from each participant's gameplay, we can explicitly calculate this opportunity cost to describe the economic motivations for breaking compliance. On average participants earned $18.29 (σ = *$*2.92) simulation dollars per round when using the shower-in, shower-out biosecurity practice, and $22.42 (σ = *$*3.56) when using the emergency exit, if their animals did not become sick, equating to an estimated $4.13 opportunity cost for using the biosecurity practice. This accounts to approximately $99.12 simulation dollars ($0.28 USD) over the course of the 24 experimental scenarios, or “rounds” of gameplay. However, this opportunity cost was contrasted with the monetary penalty of contracting an infection. If the player's facility became infected, the round ended, and they incurred a monetary penalty ($50 simulation dollars) and lost any experimental dollars they had accrued during that round. Thus, players were expected to balance the economic tradeoff regarding completing their tasks quickly but with the risk of contaminating their facility, or completing their tasks more slowly (opportunity costs) yet safely. At the end of each round, or simulated workday, the next round begins with a new set of experimental treatment parameters. This continues until all 24 rounds of gameplay are completed.

Each simulation round was designed to test how participants will behave under a set of treatments, or initialization factors, which act as our experimental variables. This allows us to quantify behavior with respect to a set of predefined epidemiological parameters and interface modifications to compare rates of compliance. The risk communication prompts included one of three types of messages describing the risk of infection associated with non-compliance with the shower protocol: 1) a linguistic (i.e., Very Low, Low, Medium, High) infection probability message, 2) a graphical threat gauge (see Figure 1), or 3) a discrete numeric or percentile value (i.e., 25%). These messages corresponded to four probabilities of infection if participants used the emergency exit: We tested a 1% (Very Low), 5% (Low), 15% (Medium), and 25% (High) probability of infection. Uncertainty in the decision making process was also tested by specifying that the disease was described as having known infection or contagion rates, or unknown characteristics prompting estimates of infection or contagion rates. If the disease was described as unknown then a range of contagion risk rates (e.g., infection risk is very low to medium with a best estimate of low risk) was provided instead of a discrete value.

The focus of the experimental simulation was to observe a participant's willingness to comply with the shower biosecurity protocol throughout various risk messaging strategies. Behavioral risk was quantified using the rate of compliance throughout the experimental simulation. This was calculated as the proportion of shower exits throughout each participant's gameplay. We then normalized by the total number of decisions to obtain each participant's risk score on a scale of 0 to 1: with 0 being risky (all emergency exits) and 1 as risk averse (all shower exits). The coin collection (indoor tasks) were designed to immerse participants within the simulated decision mechanism, so these were not pertinent for quantifying risk.

To quantify behavioral strategies within the experimental game, we applied the K-means clustering algorithm (24) to the observed rates of compliance for each participant. We administered K-means to the one-dimensional set of observations calculated using the average rate of compliance across all 24 experimental scenarios. Although we could have split these observations by treatment in order to perform a multi-dimensional analysis, we found the one dimensional case produced a straightforward distribution of behavioral strategies. We chose *K* = 3, using the elbow method by inspection, as this value optimized the sum of the square errors across each clustered interval (25, 26) and generated a rational division of observed behavioral compliance.

**Figure 2:**
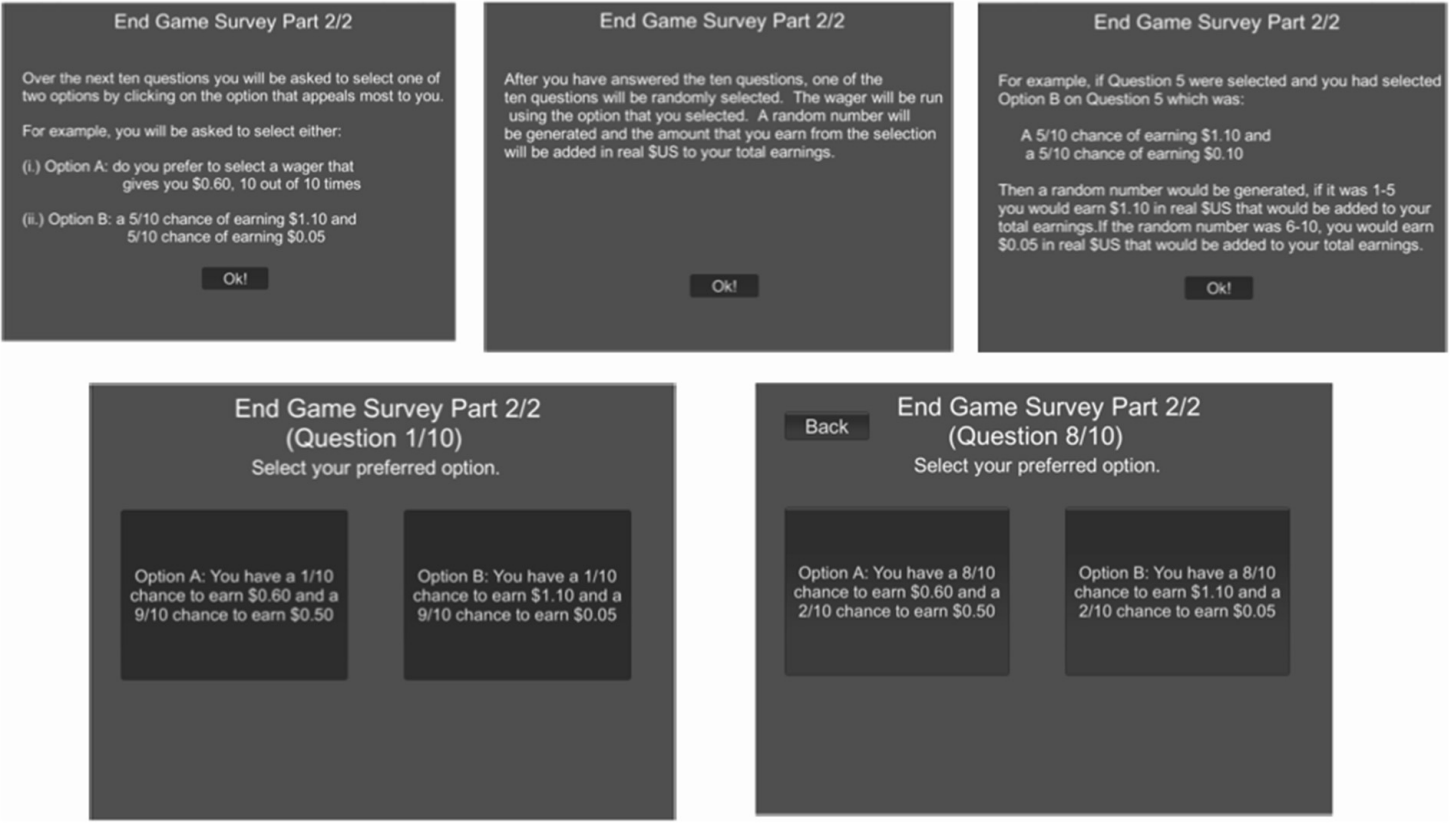
Multiple price list (MPL) survey structure.

Upon completing the experimental simulation, participants were directed to an exit survey containing a demographic questionnaire and the MPL assessment derived from Holt ann Laury (16). Participants were informed that their choices in the experimental lottery and resultant winnings would be added to their final compensation. Figure 2 provides the MPL directions and interface. Here, participants were instructed to choose their preference across ten disjoint paired lotteries: a safer “Option A” vs. a more risky “Option B.” The payout between option A is either $0.60 or $0.50 USD while the payout for the more risky Option B was $1.10 or $0.05. For example, in the first choice both “Option A” and “Option B” have a 1/10 chance for the high payout and 9/10 chance for the low payout. Here, it best to choose Option A, as Option B has a 90% chance at only earning a nickel. The probability for the high payout increases by 10% sequentially per lottery question. This makes the risky “Option B” more favorable toward the end of the survey. Participants at some point generally crossover between the safe Option A and more risky Option B, as the probability spread becomes more favorable. The final question features a 100% chance for the high payout, in which “Option B” becomes the optimal and rationally should be chosen over “Option A.” Participants could revise their decision up until their final choice, after which a random number generator selected one of their choices and issued their corresponding reward. This payout is then added to their experimental game earnings.

Risk in the MPL was measured using the proportion of safe “Option A” choices throughout the survey. Following, Holt and Laury (16) participants with 4 out of 10 safe choices were classified as risk neutral; more than 4 safe choices were classified as risk averse; and less than 4 safe choices were considered risk tolerant. We excluded participants who didn't choose at least one safe and one risky option during the MPL portion of the experiment, as this was indicative of a lack of understanding of the paired lottery choice mechanics or a sign of potential survey fatigue. Data from these participants were also excluded from the experimental game analysis.

The goal of our study was to compare risk classifications using the experimental game and the MPL risk assessment. In particular we aimed to answer the question: Does more compliance in the experimental game correspond to more risk aversion in the MPL? To compare distributions of risk between the experimental game and MPL, we used standard Pearson linear correlation (27). We compared distributions of simulated compliance and MPL risk assessment using Mann Whitney (MW) U-tests (28). We chose a non-parametric test, as both distributions of compliance and MPL risk failed D'Agostino and Pearson's test for normality (29, 30). This allowed us to infer whether groups of participants categorized by the MPL as risk tolerant, risk neutral, or risk averse complied more or less with the simulated biosecurity shower protocol. We also compared the categorical distributions from the clustering analysis of game data to MPL risk classification using a Chi Square (χ^2^) test (31). The strength of this association was then quantified with Cramer (32), which is measured on a scale of 0 (no association) to 1 (strong association). Statistics were calculated using Python (v3) with the SciPy stats module (33).

## 3. Results

We recruited 1,284 participants using the MTurk online survey marketplace between March 5th and March 31st, 2021. Participants were compensated with a base wage of $3.00 for completing the task and earned an average bonus of $2.72 which included their game performance and MPL earnings. The median completion time for both simulation and survey was 32 min.

**Figure 3:**
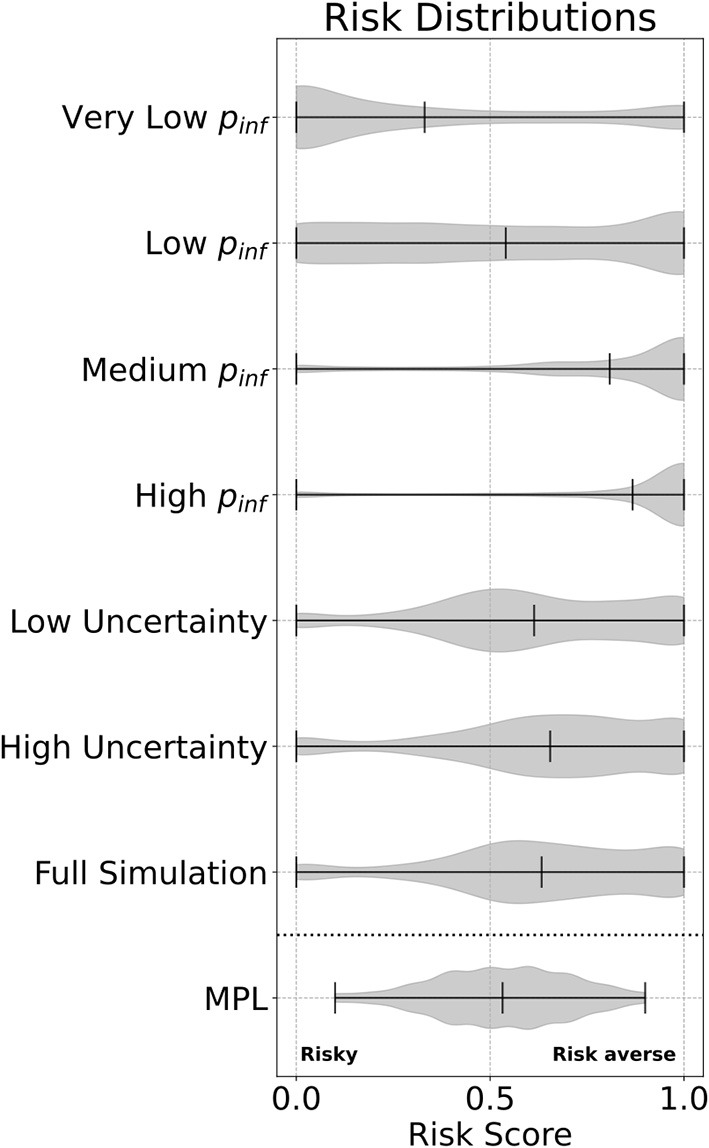
Violin plots showing risk distributions calculated for each experimental game treatment: probability of infection (*p*_*inf*_) and disease information uncertainty. The overall risk observed from the full simulation (i.e., all experimental scenarios) as well as the MPL calculated risk are given in the final violin plots.

In both the simulation and the MPL assessment, participant choices were generally skewed toward more risk averse behaviors, which in the case of the MPL is supported by previous research (16). Participants in the MPL were classified as 67.2% risk averse, 17.46% neutral, and 15.41% risk tolerant. Overall, participants complied with the shower-in, shower-out biosecurity practice on average 63% of their game play. Within the simulation, the communicated infection probability had the strongest impact on compliance with the biosecurity practices, with 87% average compliance during rounds where the probability of infection was high vs. 81% during the medium infection probability rounds, 54% during low infection probability rounds, and 33% during very low probability rounds. Risk distributions for each set of experimental treatments as well as the MPL assessment are given in Figure 3.

To quantify behavioral strategies within the experimental game, we applied the K-means clustering algorithm with *K* = 3. Cluster 1 contained the most *risk averse* participants (38%), who complied most often with the shower protocol throughout each experimental scenario. In particular, these participants had the highest compliance rate even when the probability of infection was both “Low” and “Very Low.” Cluster 2 consisted of the most *risk tolerant* individuals (13.8%). They had the least rate of compliance throughout all experimental scenarios. Participants in cluster 3, which we refer to as *opportunists* (48.2%), adapted their behavior depending on the risk of infection. During “Medium” and “High” risk rounds, they complied more often with the shower protocol yet took more risks during rounds with “Low” and “Very Low” probabilities of infection. The rates of compliance of each behavioral cluster for each treatment are given in Table 1.

**Table 1:** Experimental game risk cluster compliance comparison.

	**Cluster 1**	**Cluster 2**	**Cluster 3**
	**(Risk averse)**	**(Risk tolerant)**	**(Opportunist)**
	**(*n* = 444)**	**(*n* = 161)**	**(*n* = 563)**
Very Low *p*_*inf*_	0.71 (0.3)	0.06 (0.13)	0.11 (0.19)
Low *p*_*inf*_	0.92 (0.13)	0.08 (0.14)	0.37 (0.23)
Medium *p*_*inf*_	0.98 (0.07)	0.18 (0.22)	0.84 (0.19)
High *p*_*inf*_	0.99 (0.05)	0.29 (0.32)	0.93 (0.16)
Low Uncertainty	0.89 (0.11)	0.15 (0.14)	0.52 (0.11)
High Uncertainty	0.90 (0.11)	0.15 (0.16)	0.60 (0.13)
Full Simulation	0.90 (0.09)	0.15 (0.13)	0.56 (0.10)

Average rates of compliance (and standard deviations) are given for each set of experimental treatments per risk cluster.

We found very weak positive correlation between the compliance rate within the experimental simulation and the MPL risk assessment (Pearson *r* = 0.203, *p* < 0.001, *N* = 1, 168) . Risk measured using the MPL was not a strong predictor of participant's behavior in the experimental game. That is, a participant's rate of compliance in the experimental game did not directly relate to their risk aversion score within the MPL. In this way, we could not adequately determine if a participant may comply more or less with the shower protocol given their choices within the MPL.

Participants who did not choose both the low and high risk option at least once during the MPL assessment were excluded from the analysis. However, including the data of these 116 participants (9.03% of the sample) would not have changed the overall findings of our statistical analysis. Specifically, the correlation between the simulation and MPL risk aversion metric was still positive, yet slightly lower (Pearson *r* = 0.173, *p* < 0.001, *N* = 1, 284).

We also investigated how compliance rates differed when grouping participants by their risk classification within the MPL. In other words, we compiled rates of compliance from participant's classified as risk tolerant, neutral, and risk averse in the MPL to compare whether or not they, as a group, behaved differently within the game. We found that participants classified as risk averse *via* the MPL, complied more often with the biosecurity shower protocol vs. those classified as risk tolerant (MW: *U* = 85, 874.5;*p* < 0.001). Risk neutral participants also complied with the shower protocol less than the risk averse group (MW: *U* = 90, 655.0, *p* < 0.01) and more often than the risk tolerant group (MW: *U* = 16524.5, *p* < 0.05). These results are summed up in Table 2. This shows that the MPL classification can be useful to capture group behavioral dynamics (i.e., belonging to one of these groups may allow us to infer whether the individual may be more prone to risky behaviors comparatively).

**Table 2:** Experimental game average compliance rates stratified by MPL risk classification (*N* = 1,168).

**MPL**	**Average game**	**Standard**	***n* (%)**
**classification**	**compliance**	**deviation**	
Risk Averse	0.66	0.25	784 (67.12%)
Neutral	0.60	0.27	204 (17.46%)
Risk Tolerant	0.55	0.31	180 (15.41%)

Finally, we compare the 3 risk clusters found within the experimental game to the MPL risk categories: Risk Averse, Neutral, and Risk Tolerant. Using a Chi-Square test, we found that these categorical distributions were related [χ^2^ = 47.33, *p* < 0.0001 ]. To measure the strength of the association between distributions we calculated Cramér's V as 0.142. This shows that although these categorical distributions are related, the association is relatively weak. The contingency table for each of the observed counts of these categorical distributions is given in Table 3.

**Table 3:** Contingency Table comparing participants who were categorized using the game data clustering analysis (Rows) and classification using the MPL assessment (columns).

		**MPL**	**Risk**	**Assessment**
		**Risk tolerant**	**Neutral**	**Risk averse**	**Total**
Experimental	Cluster 1 (Risk Averse)	52 (11.7%)	62 (14.0%)	330 (74.3%)	444
Game	Cluster 2 (Risk Tolerant)	50 (31.0%)	33 (20.5%)	78 (48.4%)	161
Clusters	Cluster 3 (Opportunist)	78 (13.9%)	109 (19.4%)	376 (66.8%)	563
	Total	180	204	784	1,168

For each observed frequency, the percentage of the category (row) count is given in parentheses.

## 4. Discussion and conclusion

Experimental gaming simulations can provide a unique lens for quantifying behavioral risk. Contextual framing allows for these simulations to elicit particular behaviors of interest in comparison to generalized survey methods. Combining economic risk aversion metrics derived from traditional survey methodologies along with behaviors captured *via* simulation may help build risk profiles that better capture how human behavior may impact the resiliency of these systems.

Risk communication strategies are important tools for mitigating disease spread among other crisis situations (13). Experimental games are well suited to test the influence of risk communication strategies on behavior in these types of contrived instances. Our simulations have shown that the type of risk communication strategy can have a large impact on behavior (7). Our clustering analysis identified the most prominent behavioral strategies driving non-compliance. Participants classified as risk averse complied with the shower protocol most often, while the risk tolerant and opportunists took more liberties with risking infection for a potential higher payout. All groups increased their compliance with the communicated probability of infection, albeit at different rates. The risk averse cluster bolstered very high rates of compliance, even when the risk of infection was very low. The risk tolerant were the least responsive to risk messaging, exhibiting high rates of non-compliance even during scenarios with high rates of infection. The opportunists were very responsive to the risk messaging and changed their strategy based upon the rate of contagion; adopting more risk as the probability of infection decreased. Experimental games can be used to identify how risk messaging impacts behavior and can help us adjust communication strategies accordingly. These malleable, digital tools are well suited for observing behaviors that may be difficult to capture in the real-world.

MPL risk assessment was not a strong predictor of simulated biosecurity compliance at the individual level. However, we did find when grouping participants *via* their MPL assessment, the risk averse participants complied with the biosecurity shower-in, shower-out practice more often than those classified as risk tolerant. We also found that the compliance rate of MPL-classified risk neutral participants was sandwiched between the risk averse and risk tolerant participants' rate of compliance observed during game play. This is interesting as it indicates that a portion of the behavioral signal regarding biosecurity compliance is preserved within the MPL assessment. It is also noteworthy that the MPL risk assessment can be administered without computers and thus may presently be better equipped for sampling low income populations, especially in countries lacking internet access. This shows, from a broad perspective, the MPL risk aversion assessment can be a useful option as it is lightweight and easily deployable in comparison to experimental games.

We should also note that the MPL risk assessment was administered directly after the experimental game. It is possible that the experience within the game may have had a priming effect on our participants. Though in our case, the MPL and experimental game were very different thematically; one testing pure economic risk aversion while the other simulated risk associated with animal disease spread. It is still possible that the profits and losses experienced within the game could have had some influence on the behavioral risk exhibited within the MPL. However, since the risk distributions of the MPL were found to align with previous research, this suggests the order of administration did not have a large impact on our findings, but should be considered in future experiments.

Although MPL risk assessment has many useful applications, the contextual framing provided by experimental games may be better equipped for targeting situational risk and capturing nuanced behaviors at the individual level. Our results show that group behaviors may be inferred using the MPL classification, however could not be reliably applied to the individual. This may suggest that pure economic risk does not fully translate to situational risk, even when the monetary drivers may be comparable. This may be a consequence of the contextual framing embedded within the experimental game. One's actions within the simulated world had an effect on the well-being of a farm and livestock health. This immersion factor may have played a role in motivating individuals to behave differently as opposed to purely economic motivators to achieve a higher payout. This substantiates the use of experimental games for studying complex decision mechanisms and for building circumstantial risk profiles. Identifying behavioral risk in this way can help us better understand the decision-making process, and allow us to study how best to intervene or nudge human behavior toward safer practices.

Identifying risk profiles is pertinent for modeling disease spread across agricultural production networks. Agent based modeling approaches (34–37) can be implemented to simulate disease spread scenarios and economic impacts across supply chain networks (38). Incorporating human behavior components into these models can vastly impact the projections of disease spread (39). Experimental simulations can help inform these models to make more realistic epidemiological forecasts. In particular, using outcomes from experimental games (i.e., rates of compliance) to inform modeling approaches can be useful in projecting potential damages to the supply chain. Within the model, agents' decision heuristics can be adapted from behavioral strategies observed within the experimental game. Experimental games can help us identify realistic and prominent behavioral strategies along with distributions of risk profiles to parameterize modeling initiatives. This can help industry professionals account for these outcomes and work toward strengthening our systems against disease spread.

Quantifying emergent strategies using experimental games can help us better understand the broad behaviors most prominently driving outcomes within these scenarios. Identifying a risk metric, in our case rates of biosecurity compliance, and then applying a clustering algorithm can be a straightforward solution for quickly identifying prominent behaviors. Here, we found three overall strategies: risk tolerant, risk averse, and opportunist. Interestingly, we found the same categorical behaviors from another experimental game that focuses on managing a pig farm's biosecurity investment during various outbreak scenarios (9). Although mechanically these games were very different, as were the risk metric and clustering strategy, the emergent group behaviors were analogous. In the biosecurity adoption game, the risk tolerant invested very little in biosecurity across all scenarios; the risk averse invested the most in biosecurity throughout, and the opportunists changed their investment strategy based upon the rate of contagion. These three broad behavioral groups are thematic and can likely can be applied to a wide variety of decision mechanisms. Identifying membership within these groups can help us work toward individualizing our risk messaging strategies and our ability to influence positive changes in behavior.

Our study supports using experimental games for studying contextualized risk situations. These digital tools can provide nuanced insight into how context influences risk aversion using immersive, complex environments to explore adaptive and dynamic decision-making. Experimental games have a large potential for expanding upon traditional survey instruments by enveloping participants within a complex decision mechanism, allowing us to capture dynamic and evolving behavioral signals. By combining these simulations with survey instruments, we can work toward generating stronger risk profiles with more predictive power for modeling how human behavior impacts crisis situations. More research should be conducted to explore how experimental gaming simulations can be used for quantifying behavior and the resultant impact on the well-being of our agricultural industry. These digital applications can provide valuable insights that may allow us to nudge social ecological systems, such as swine production, to improve our ability to keep our systems disease resilient and promote herd health.

## Data availability statement

The raw data supporting the conclusions of this article will be made available by the authors, without undue reservation.

## Ethics statement

The studies involving human participants were reviewed and approved by the University of Vermont Institutional Review Board concerning experiments using human participants (University of Vermont IRB # CHRBSS-16-232-IRB). Written informed consent for participation was not required for this study in accordance with the national legislation and the institutional requirements.

## Author contributions

EC contributed to conceptualization, data curation, formal analysis, software, visualization, and writing—original draft. SM contributed to conceptualization, investigation, methodology, project administration, supervision, and writing—review and editing. LT contributed to software and writing—review and editing. T-LL, AO'K, GB, NC, OL-C, and TS contributed to review and editing. CK contributed to funding acquisition, project administration, and writing—review and editing. AZ contributed to conceptualization and writing—review and editing and funding acquisition. JS contributed to investigation, project administration, funding acquisition, and writing—review and editing. All authors contributed to the article and approved the submitted version.

## Funding

This work was supported by the USDA National Institute of Food and Agriculture, under Award No. 2015-69004-23273 and USDA NIFA Award No. 2021-67015-35236.

## Conflict of interest

The authors declare that the research was conducted in the absence of any commercial or financial relationships that could be construed as a potential conflict of interest.

## Publisher's note

All claims expressed in this article are solely those of the authors and do not necessarily represent those of their affiliated organizations, or those of the publisher, the editors and the reviewers. Any product that may be evaluated in this article, or claim that may be made by its manufacturer, is not guaranteed or endorsed by the publisher.

## Author disclaimer

The contents are solely the responsibility of the authors and do not necessarily represent the official views of the USDA or NIFA.
